# Antagonism to the American Medical Association

**Published:** 1917-06

**Authors:** 


### Antagonism to the American Medical Association.

We note with considerable regret, in several of our exchanges, either on the part of editors or contributors, a hostile spirit toward the A. M. A. Some even went so far as to gloat over the diminished attendance at Los Angeles—a perfectly natural result of mileage and distribution of population—as an indication of loss of prestige. Subsequent experience has, of course, modified whatever force the criticisms may have had at the time. There is vague hinting at graft, talk of machine rule, criticism of the past conduct of men in power, complaint of the space and expense devoted to attacks on proprietary medicine, objection to various details but, fortunately, little or no open charge of present mismanagement.

The writer's first A. M. A. meeting was at Nashville, in 1890; he was one of the members to whom the revised system of government was submitted in 1901; one of the little group of those affiliated with the Medical Society of the State of New York who attended the Saratoga meeting in 1902 and received much individual courtesy and the official cold shoulder; one of the increasing party in the said Society to advocate reunion of the profession at whatever cost. Without wishing to appear ungracious, the opinion may be expressed that this cost has been the sacrifice of practically every characteristic of the Society save its name and hundred odd years of existence. And, in all fairness, it must be acknowledged that the wonderfully complete system of national, state and local organization achieved, the growth of the organized profession in numbers and influence, the higher grade of scientific work established for meetings, the control of medical education and numerous other benefits, have outweighed all preferences of methods and detailed policy.

We bespeak, therefore, the loyal support of the A. M. A. and of its secondary and tertiary branches and we hope that every reader will realize that both his self interest and his duty as a member of a profession and a citizen of potential influence are only half fulfilled unless he is enrolled in the respective organizations. Without such enrollment, he misses many opportunities for improvement in his art, is deprived of many benefits of a social and practical nature, and his influence on public health, professional improvement and legislation becomes almost nil.

At the same time, without disloyalty or dissatisfaction with existing conditions, it is worth while to regard the attacks upon the present system of organization, as warnings of what might, under certain conditions, become a serious menace to the united medical profession. There are a great many points of issue, as for instance, the organization of a state society as a permanent senate with membership to be earned by preliminary service as delegate, rather than on the ipso facto basis of county membership, which involve no matter of principle, but in regard to which, local opinion and precedent might well be consulted. In general, it seems that the bond of union would be no weaker if more elastic.

Those of us who remember the general meetings of the A. M. A. and of the State Society, who have been guests of the Canadian Medical Association, etc., also realize the advantages of small membership allowing a purely democratic, instead of a more or less representative form of government. The A. M. A. has outgrown the feasibility of government by the open session of members, yet we miss the active participation of the delegates in strictly medical debates, and many members and local organizations complain of the establishment of a superior governing body. It would seem possible to introduce the principle of the referendum into the government of the A. M. A. and to allow the initiative of legislation, at least by suggestion, at the hands of every member in good standing or by resolution of any component organization. A great deal of the dissatisfaction at present expressed could be dissipated if there were a safety valve for the free expression of opinions, verbally or in print, in such a way as at least to put them before the membership of the Association. And we are optimistic enough to believe that such return to democratic principles would be of value in more ways than as a safety valve.

A matter that deserves serious consideration, is the fact that the A. M. A. is, from various sources, gradually piling up a surplus of no small magnitude. With every confidence in the present management, there is ample precedent in history for expressing the fear that some time, great wealth and great power may prove to be a temptation to unwise use of these levers, even if personal gain is not attempted. It is already time to give careful attention to this problem, if for no other reason than that its mere presence affords an excuse for hostility.

